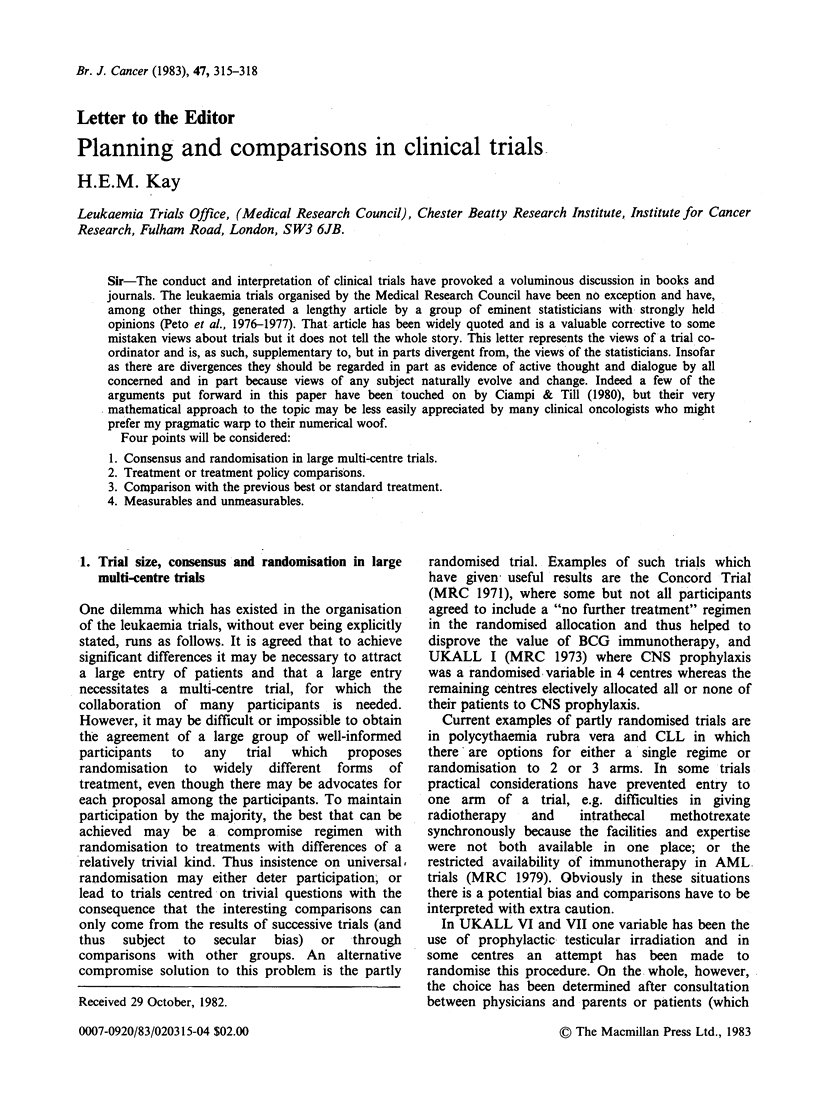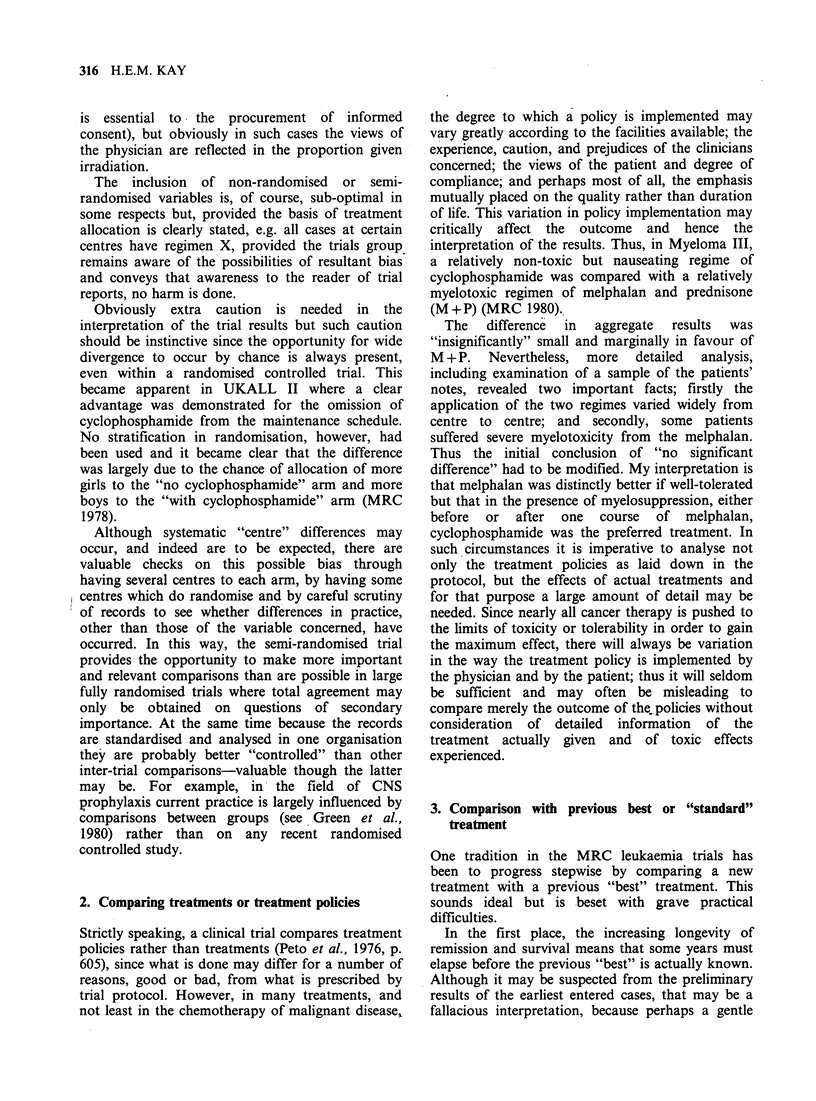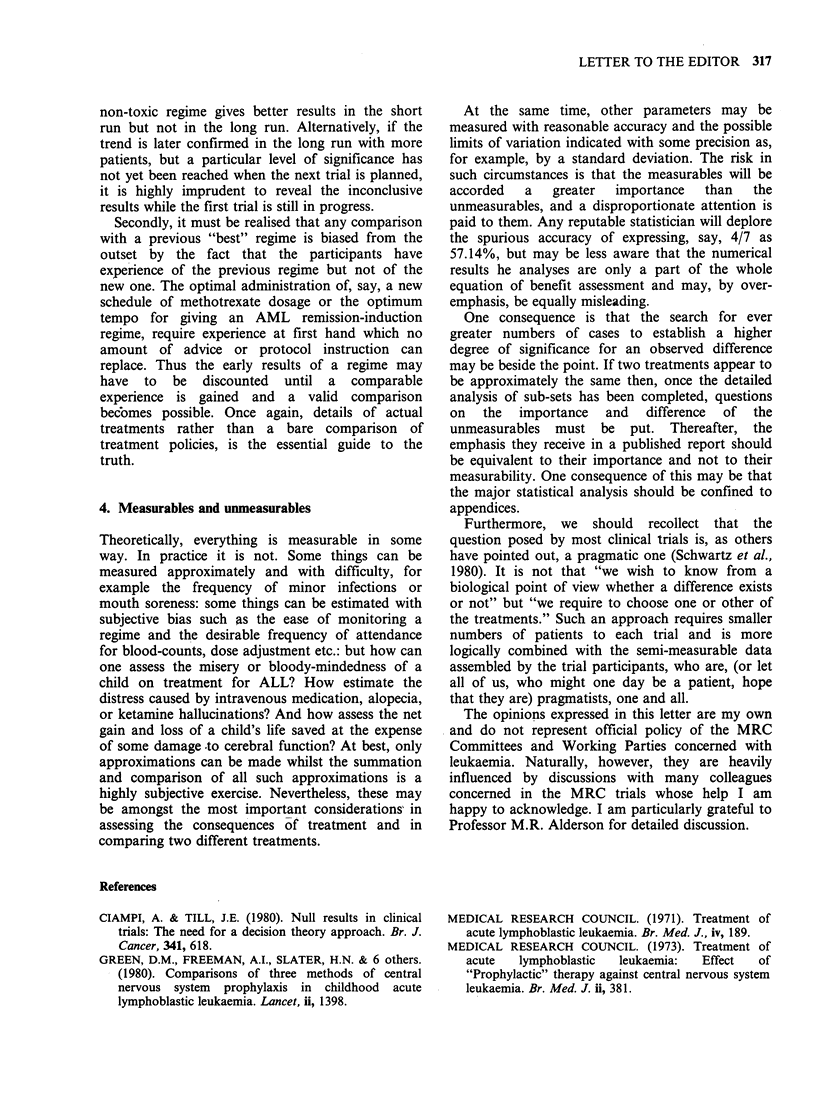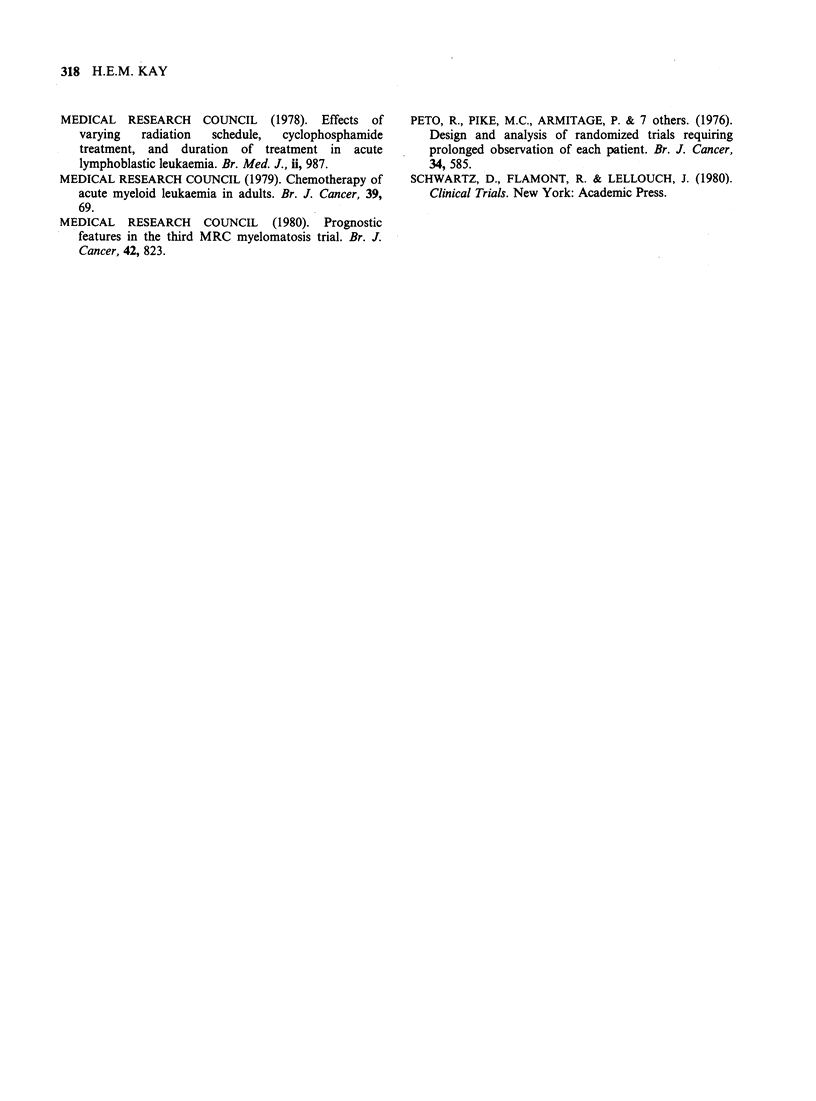# Planning and comparisons in clinical trials.

**DOI:** 10.1038/bjc.1983.43

**Published:** 1983-02

**Authors:** H. E. Kay


Br. J. Cancer (1983), 47, 315–318

### Letter to the Editor

### Planning and comparisons in clinical trials

H.E.M. Kay

*Leukaemia Trials Office, (Medical Research Council), Chester Beatty Research Institute, Institute for Cancer Research, Fulham Road, London, SW3 6JB.*

**Sir**—The conduct and interpretation of clinical trials have provoked a voluminous discussion in books and journals. The leukaemia trials organised by the Medical Research Council have been no exception and have, among other things, generated a lengthy article by a group of eminent statisticians with strongly held opinions (Peto *et al.*, 1976–1977). That article has been widely quoted and is a valuable corrective to some mistaken views about trials but it does not tell the whole story. This letter represents the views of a trial co-ordinator and is, as such, supplementary to, but in parts divergent from, the views of the statisticians. Insofar as there are divergences they should be regarded in part as evidence of active thought and dialogue by all concerned and in part because views of any subject naturally evolve and change. Indeed a few of the arguments put forward in this paper have been touched on by Ciampi & Till (1980), but their very mathematical approach to the topic may be less easily appreciated by many clinical oncologists who might prefer my pragmatic warp to their numerical woof.

Four points will be considered:

1. Consensus and randomisation in large multi-centre trials.
2. Treatment or treatment policy comparisons.
3. Comparison with the previous best or standard treatment.
4. Measurables and unmeasurables.

### 1. Trial size, consensus and randomisation in large multi-centre trials

One dilemma which has existed in the organisation of the leukaemia trials, without ever being explicitly stated, runs as follows. It is agreed that to achieve significant differences it may be necessary to attract a large entry of patients and that a large entry necessitates a multi-centre trial, for which the collaboration of many participants is needed. However, it may be difficult or impossible to obtain the agreement of a large group of well-informed participants to any trial which proposes randomisation to widely different forms of treatment, even though there may be advocates for each proposal among the participants. To maintain participation by the majority, the best that can be achieved may be a compromise regimen with randomisation to treatments with differences of a relatively trivial kind. Thus insistence on universal randomisation may either deter participation, or lead to trials centred on trivial questions with the consequence that the interesting comparisons can only come from the results of successive trials (and thus subject to secular bias) or through comparisons with other groups. An alternative compromise solution to this problem is the partly randomised trial. Examples of such trials which have given useful results are the Concord Trial (MRC 1971), where some but not all participants agreed to include a "no further treatment" regimen in the randomised allocation and thus helped to disprove the value of BCG immunotherapy, and UKALL I (MRC 1973) where CNS prophylaxis was a randomised variable in 4 centres whereas the remaining centres electively allocated all or none of their patients to CNS prophylaxis.

Current examples of partly randomised trials are in polycythaemia rubra vera and CLL in which there are options for either a single regime or randomisation to 2 or 3 arms. In some trials practical considerations have prevented entry to one arm of a trial, e.g. difficulties in giving radiotherapy and intrathecal methotrexate synchronously because the facilities and expertise were not both available in one place; or the restricted availability of immunotherapy in AML trials (MRC 1979). Obviously in these situations there is a potential bias and comparisons have to be interpreted with extra caution.

In UKALL VI and VII one variable has been the use of prophylactic testicular irradiation and in some centres an attempt has been made to randomise this procedure. On the whole, however, the choice has been determined after consultation between physicians and parents or patients (which

Received 29 October, 1982.

0007-0920/83/020315-04 $02.00 © The Macmillan Press Ltd., 1983

is essential to the procurement of informed consent), but obviously in such cases the views of the physician are reflected in the proportion given irradiation.

The inclusion of non-randomised or semi-randomised variables is, of course, sub-optimal in some respects but, provided the basis of treatment allocation is clearly stated, e.g. all cases at certain centres have regimen X, provided the trials group remains aware of the possibilities of resultant bias and conveys that awareness to the reader of trial reports, no harm is done.

Obviously extra caution is needed in the interpretation of the trial results but such caution should be instinctive since the opportunity for wide divergence to occur by chance is always present, even within a randomised controlled trial. This became apparent in UKALL II where a clear advantage was demonstrated for the omission of cyclophosphamide from the maintenance schedule. No stratification in randomisation, however, had been used and it became clear that the difference was largely due to the chance of allocation of more girls to the "no cyclophosphamide" arm and more boys to the "with cyclophosphamide" arm (MRC 1978).

Although systematic "centre" differences may occur, and indeed are to be expected, there are valuable checks on this possible bias through having several centres to each arm, by having some centres which do randomise and by careful scrutiny of records to see whether differences in practice, other than those of the variable concerned, have occurred. In this way, the semi-randomised trial provides the opportunity to make more important and relevant comparisons than are possible in large fully randomised trials where total agreement may only be obtained on questions of secondary importance. At the same time because the records are standardised and analysed in one organisation they are probably better "controlled" than other inter-trial comparisons—valuable though the latter may be. For example, in the field of CNS prophylaxis current practice is largely influenced by comparisons between groups (see Green *et al.*, 1980) rather than on any recent randomised controlled study.

### 2. Comparing treatments or treatment policies

Strictly speaking, a clinical trial compares treatment policies rather than treatments (Peto *et al.*, 1976, p. 605), since what is done may differ for a number of reasons, good or bad, from what is prescribed by trial protocol. However, in many treatments, and not least in the chemotherapy of malignant disease, the degree to which a policy is implemented may vary greatly according to the facilities available; the experience, caution, and prejudices of the clinicians concerned; the views of the patient and degree of compliance; and perhaps most of all, the emphasis mutually placed on the quality rather than duration of life. This variation in policy implementation may critically affect the outcome and hence the interpretation of the results. Thus, in Myeloma III, a relatively non-toxic but nauseating regime of cyclophosphamide was compared with a relatively myelotoxic regimen of melphalan and prednisone (M+P) (MRC 1980).

The difference in aggregate results was "insignificantly" small and marginally in favour of M+P. Nevertheless, more detailed analysis, including examination of a sample of the patients' notes, revealed two important facts; firstly the application of the two regimes varied widely from centre to centre; and secondly, some patients suffered severe myelotoxicity from the melphalan. Thus the initial conclusion of "no significant difference" had to be modified. My interpretation is that melphalan was distinctly better if well-tolerated but that in the presence of myelosuppression, either before or after one course of melphalan, cyclophosphamide was the preferred treatment. In such circumstances it is imperative to analyse not only the treatment policies as laid down in the protocol, but the effects of actual treatments and for that purpose a large amount of detail may be needed. Since nearly all cancer therapy is pushed to the limits of toxicity or tolerability in order to gain the maximum effect, there will always be variation in the way the treatment policy is implemented by the physician and by the patient; thus it will seldom be sufficient and may often be misleading to compare merely the outcome of the policies without consideration of detailed information of the treatment actually given and of toxic effects experienced.

### 3. Comparison with previous best or "standard" treatment

One tradition in the MRC leukaemia trials has been to progress stepwise by comparing a new treatment with a previous "best" treatment. This sounds ideal but is beset with grave practical difficulties.

In the first place, the increasing longevity of remission and survival means that some years must elapse before the previous "best" is actually known. Although it may be suspected from the preliminary results of the earliest entered cases, that may be a fallacious interpretation, because perhaps a gentle non-toxic regime gives better results in the short run but not in the long run. Alternatively, if the trend is later confirmed in the long run with more patients, but a particular level of significance has not yet been reached when the next trial is planned, it is highly imprudent to reveal the inconclusive results while the first trial is still in progress.

Secondly, it must be realised that any comparison with a previous "best" regime is biased from the outset by the fact that the participants have experience of the previous regime but not of the new one. The optimal administration of, say, a new schedule of methotrexate dosage or the optimum tempo for giving an AML remission-induction regime, require experience at first hand which no amount of advice or protocol instruction can replace. Thus the early results of a regime may have to be discounted until a comparable experience is gained and a valid comparison becomes possible. Once again, details of actual treatments rather than a bare comparison of treatment policies, is the essential guide to the truth.

### 4. Measurables and unmeasurables

Theoretically, everything is measurable in some way. In practice it is not. Some things can be measured approximately and with difficulty, for example the frequency of minor infections or mouth soreness: some things can be estimated with subjective bias such as the ease of monitoring a regime and the desirable frequency of attendance for blood-counts, dose adjustment etc.: but how can one assess the misery or bloody-mindedness of a child on treatment for ALL? How estimate the distress caused by intravenous medication, alopecia, or ketamine hallucinations? And how assess the net gain and loss of a child's life saved at the expense of some damage to cerebral function? At best, only approximations can be made whilst the summation and comparison of all such approximations is a highly subjective exercise. Nevertheless, these may be amongst the most important considerations in assessing the consequences of treatment and in comparing two different treatments.

At the same time, other parameters may be measured with reasonable accuracy and the possible limits of variation indicated with some precision as, for example, by a standard deviation. The risk in such circumstances is that the measurables will be accorded a greater importance than the unmeasurables, and a disproportionate attention is paid to them. Any reputable statistician will deplore the spurious accuracy of expressing, say, 4/7 as 57.14%, but may be less aware that the numerical results he analyses are only a part of the whole equation of benefit assessment and may, by over-emphasis, be equally misleading.

One consequence is that the search for ever greater numbers of cases to establish a higher degree of significance for an observed difference may be beside the point. If two treatments appear to be approximately the same then, once the detailed analysis of sub-sets has been completed, questions on the importance and difference of the unmeasurables must be put. Thereafter, the emphasis they receive in a published report should be equivalent to their importance and not to their measurability. One consequence of this may be that the major statistical analysis should be confined to appendices.

Furthermore, we should recollect that the question posed by most clinical trials is, as others have pointed out, a pragmatic one (Schwartz *et al.*, 1980). It is not that "we wish to know from a biological point of view whether a difference exists or not" but "we require to choose one or other of the treatments." Such an approach requires smaller numbers of patients to each trial and is more logically combined with the semi-measurable data assembled by the trial participants, who are, (or let all of us, who might one day be a patient, hope that they are) pragmatists, one and all.

The opinions expressed in this letter are my own and do not represent official policy of the MRC Committees and Working Parties concerned with leukaemia. Naturally, however, they are heavily influenced by discussions with many colleagues concerned in the MRC trials whose help I am happy to acknowledge. I am particularly grateful to Professor M.R. Alderson for detailed discussion.

### References

CIAMPI, A. & TILL, J.E. (1980). Null results in clinical trials: The need for a decision theory approach. *Br. J. Cancer*, **341**, 618.

GREEN, D.M., FREEMAN, A.I., SLATER, H.N. & 6 others. (1980). Comparisons of three methods of central nervous system prophylaxis in childhood acute lymphoblastic leukaemia. *Lancet*, **ii**, 1398.

MEDICAL RESEARCH COUNCIL. (1971). Treatment of acute lymphoblastic leukaemia. *Br. Med. J.*, **iv**, 189.

MEDICAL RESEARCH COUNCIL. (1973). Treatment of acute lymphoblastic leukaemia: Effect of "Prophylactic" therapy against central nervous system leukaemia. *Br. Med. J.* **ii**, 381.

MEDICAL RESEARCH COUNCIL (1978). Effects of varying radiation schedule, cyclophosphamide treatment, and duration of treatment in acute lymphoblastic leukaemia. *Br. Med. J.*, **ii**, 987.

MEDICAL RESEARCH COUNCIL (1979). Chemotherapy of acute myeloid leukaemia in adults. *Br. J. Cancer,* **39**, 69.

MEDICAL RESEARCH COUNCIL (1980). Prognostic features in the third MRC myelomatosis trial. *Br. J. Cancer,* **42**, 823.

PETO, R., PIKE, M.C., ARMITAGE, P. & 7 others. (1976). Design and analysis of randomized trials requiring prolonged observation of each patient. *Br. J. Cancer,* **34**, 585.

SCHWARTZ, D., FLAMONT, R. & LELLOUCH, J. (1980). *Clinical Trials*. New York: Academic Press.